# Peer Recovery Care Planning While Awaiting Alcohol and Other Drug Treatment

**DOI:** 10.1111/dar.70225

**Published:** 2026-07-30

**Authors:** Annette Peart, Ariel D. Roxburgh, Lauren Findlay, Benn Veenker, Tarisai Shinya, Victoria Manning, David Best, Dan I. Lubman

**Affiliations:** ^1^ Turning Point, Eastern Health Melbourne Australia; ^2^ Monash Addiction Research Centre and Eastern Health Clinical School Monash University Melbourne Australia; ^3^ Star Recovery Bamford Derbyshire UK

**Keywords:** care planning, recovery capital, strengths‐based approaches, waiting lists

## Abstract

**Introduction:**

People experiencing addiction often face barriers to care, including long wait times and social isolation. Peer support, delivered by people with lived experience, can address these challenges by fostering engagement and building recovery skills. This study piloted a peer intervention using the RECovery CAPital (REC‐CAP) strengths assessment and planning tool for individuals on a waiting list for alcohol and other drug community treatment.

**Methods:**

Eligible participants (*n* = 21) were referred to peer workers for an assessment and goal setting session using REC‐CAP between April and August 2024. Follow‐up sessions occurred at 4‐ and 12‐weeks to revisit goals and readminister REC‐CAP, alongside brief fortnightly check‐ins. At 12 weeks, qualitative interviews explored participant experiences and identified suggestions for improvement. Interviews were analysed using inductive content analysis. Changes in recovery capital (REC‐CAP score) were assessed using a general linear model.

**Results:**

Of the 21 participants (55% male, mean age 44), 72% identified alcohol as their primary drug of concern. Recovery capital significantly increased across the three time points, with the greatest gains observed between baseline and 4 weeks (*n* = 15). Qualitative findings highlighted the value of peer connection and structured goal setting while waiting for treatment.

**Discussion and Conclusions:**

Peer support based on REC‐CAP strengths profile and care planning shows promise in increasing recovery capital and maintaining motivation during treatment delays. Participants valued the peer relationship and the opportunity to reflect on recovery goals. These findings support further investigation in a fully powered trial to assess impact, effectiveness and scalability.

## 1 Introduction

For people experiencing addiction, timely access to treatment, care and support is essential to minimise harms, manage substance use and support recovery. A range of treatment options is available in Australia, including withdrawal management, pharmacotherapy and counselling. Despite this, access to addiction treatment, including counselling, remains a significant unmet need. In Australia, it is estimated that in 2022–2023, fewer than one‐third to one‐half of people who sought addiction treatment actually received it [1]. Barriers to engagement include stigma [2] and systemic factors such as longstanding underinvestment in addiction services and high attrition prior to treatment commencement due to long waiting times [3, 4, 5, 6].

The waiting period for addiction treatment has been described as the ‘dead zone’, referring to a fragmented and often prolonged experience that includes waiting for intake, referral, assessment and eventual engagement in treatment [5]. Longer waiting times are associated with a reduced likelihood of entering treatment [7] and are further compounded by systemic and individual factors, including fluctuations in motivation [8, 9]. Importantly, the experience of waiting is not uniform; for many, it can exacerbate existing barriers such as stigma and marginalisation, while also missing critical windows of readiness for change [8, 10].

Interventions aimed at addressing delays in treatment access include organisational quality improvement strategies, such as enhancing telephone scheduling processes [11], text messaging between emergency department visits and outpatient treatment engagement [12] and brief motivational telephone calls [13]. Increasingly, peer‐based recovery support (PBRS) [14], delivered by individuals with lived experience of recovery, has emerged as a promising approach to addressing access barriers and maintaining engagement during waiting periods [15]. Recovery is understood as a process of change through which individuals improve health and wellness, live a self‐directed life and strive to reach their full potential [16], and peers draw on this lived experience to support others through offering emotional support, practical assistance, information about recovery pathways and fostering social connection [17]. While research on PBRS is still emerging, evidence suggests peer interventions have a ‘salutary effect’ on participants and contribute positively to treatment and recovery outcomes [18]. This positive impact aligns with contemporary frameworks that emphasise the importance of recovery capital (encompassing social, physical, human and cultural resources) in supporting and sustaining long‐term recovery.

In recent years, addiction recovery has increasingly been conceptualised as a dynamic process supported by the development of recovery capital. Recovery capital refers to the internal and external resources a person can draw upon to initiate and sustain recovery [19, 20]. The (REC‐CAP) recovery capital is an evidence‐based assessment and recovery planning tool designed to help identify a person's strengths, barriers and unmet service needs, support goal‐setting and track recovery progress over time [21]. REC‐CAP results are used to tailor recovery‐oriented care plans and monitor longitudinal change. There is a limited, but increasing, evidence‐base supporting the use of the REC‐CAP in addiction treatment settings, in particular in drug courts, recovery housing and therapeutic communities [21, 22, 23, 24]. However, there has been no research to date examining the use of REC‐CAP as the basis for recovery care planning, both in relation to outcomes and the experience of using REC‐CAP as a peer or service recipient.

We developed a peer intervention incorporating the REC‐CAP for people on a waiting list for outpatient addiction counselling. While increasing service capacity remains the most direct way to reduce treatment delays, broader workforce and funding reforms are beyond the scope of any single service and are unlikely to be implemented quickly. Waiting times are also likely to persist while demand continues to exceed capacity, and reducing wait times alone does not address barriers such as social isolation, stigma and fluctuating motivation. The intervention was therefore designed to complement, rather than replace, broader system reforms. While acknowledging the structural barriers that limit timely access to care, the intervention was designed to enhance motivation and engagement in recovery‐oriented activities (e.g., community‐based peer self‐help groups, re‐engagement in self‐identified valued roles and activities) during the typical 12‐week waiting period. The intervention aimed to strengthen recovery capital, as measured by the REC‐CAP, during this period of otherwise limited support. Therefore, the aim of this paper is to report findings from a pilot study of this intervention, focusing on recovery capital as the primary outcome, as well as participants' (including staff) experiences of the intervention.

## 2 Methods

### 2.1 Study Design

The study was a single‐arm pilot of a peer intervention using REC‐CAP to support the Assessment of Recovery Capital (ARC) and goal setting. Quantitative and qualitative data were collected to determine preliminary effectiveness on recovery capital and participant experiences of the intervention. The study was approved by the Human Research Ethics Committees of Eastern Health (EH‐2024‐414862) and Monash University (102063).

### 2.2 Participants

Participants were individuals seeking addiction counselling within a catchment area serving the eastern suburbs of Melbourne, Australia, recruited between April and August 2024. They had either been referred or self‐referred to a centralised intake service responsible for assessing needs and connecting people with appropriate treatment providers. Participation in the study occurred in the context of voluntary treatment‐seeking. Referrals to the intake service could occur via a telephone contact with individual service providers (who directed the person to the intake service), or through DirectLine (https://www.directline.org.au/), a statewide telephone and online service that supports people seeking information, advice and referrals related to alcohol and other drugs.

Intake workers identified eligible participants. Participants were eligible if, following completion of the intake process, they were assessed as needing addiction counselling and placed on the counselling waiting list, were aged 18 years or older and had sufficient English literacy to complete the REC‐CAP. Exclusion criteria included a need for medical intervention, including withdrawal management, inpatient treatment or addiction specialist assessment.

Eligible participants received project information via email and were contacted by a researcher for verbal explanation and consent. Once consent was obtained, participant details were shared with peer workers, who arranged the first intervention session.

The two peer workers (one man, one woman) participating in the intervention were staff with lived experience of addiction, supported by formal training, who help others through authentic connection, hope and shared experience [25]. They had between one and three years' experience as peer workers.

### 2.3 Intervention

Prior to recruitment, peer workers attended a half‐day training session designed by the researchers to learn about the project, administration of REC‐CAP and the goal setting process. Training included role play and case studies to illustrate the intervention components. Intervention adherence or fidelity was not formally assessed. However, during the intervention period, peer workers participated in fortnightly Zoom sessions with researchers to provide feedback and problem‐solve issues as they arose.

The peer workers provided all components of the intervention. The intervention comprised three face‐to‐face sessions between participants and peer workers, held at the community‐based centre where intake and treatment services were provided. The three sessions were: (i) Baseline session consisting of rapport building, followed by participants collaboratively completing the REC‐CAP with the peer worker using electronic data capture software (REDCap) [26], and developing recovery‐oriented goals based in part on the results from the REC‐CAP; (ii) a 4‐week follow‐up session, focused on re‐assessing progress via REC‐CAP and reviewing goals achieved; and (iii) an optional 12‐week follow‐up session completing REC‐CAP and reviewing goals. The REC‐CAP provided a colour‐coded output of participant scores, indicating high, medium and low scoring domains of recovery capital. These scores were used by peer workers and participants to support collaborative goal setting. Peer workers were encouraged to tailor sessions to the specific needs of each participant. At the end of the 12 weeks, peers concluded their intervention with participants, at which point participants would be typically ready to commence addiction counselling and exit the waiting list.

Each session lasted approximately 2 h. In addition to these sessions, peer workers maintained fortnightly telephone contact with participants (as part of the intake service's usual care, provided by peer and non‐peer staff) while they remained on the waiting list. The Template for Intervention Description and Replication [27] for the intervention is available in Appendix A.

### 2.4 Outcomes

The Overall Recovery Capital Score (ORCS) [22] was the primary outcome measure and incorporates the majority of items within the REC‐CAP tool. REC‐CAP incorporates several validated measures including: (i) the ARC (a measure of recovery capital) [28]; (ii) the World Health Organization's Quality of Life assessment (WHOQOL‐BREF; a quality of life assessment) [29]; (iii) five ‘Barriers to Recovery’ items, adapted from the Treatment Outcome Profile (a psychological health scale) [30]; (iv) Service Involvement Needs (a measure of unmet service needs developed for the REC‐CAP) [21]; (v) the Recovery Group Participation Scale (RGPS; a measure of participation in recovery groups) [31]; and (vi) the Commitment to Sobriety Scale (CSS; a measure of a person's commitment to sobriety) [32]. The ORCS creates a total recovery capital score ranging from −100 to 100, where higher scores indicate greater recovery capital. The scores from ‘Barriers to Recovery’ and the Service Involvement Needs are combined to give a score ranging from 0 to −100 representing Negative Recovery Capital. The scores from the ARC, WHOQOL‐BREF, RGPS and CSS are combined to give a score ranging from 0 to 100 representing Positive Recovery Capital. Negative and Positive Recovery Capital scores are collated to give the total ORCS.

The REC‐CAP also included seven questions (requiring Yes/No responses) about ongoing support needs to help with goal setting. ‘Do you need help or additional help with: (1) Alcohol and Other Drug treatment; (2) Mental health; (3) Housing support; (4) Employment; (5) Primary healthcare (general practitioner, medical services); (6) Family relationships; and, (7) Other specialist help/support (please specify)’.

Outcomes were measured at 4 and 12 weeks. The primary outcome was change in ORCS between baseline and the two follow‐ups. Secondary outcomes included changes in the REC‐CAP subscales (ARC, WHOQL‐BREF, Treatment Outcome Profile, RGPS, CSS) following the intervention. Qualitative data was collected via telephone interviews at the 12‐week time point to understand participant and staff experiences of the intervention.

### 2.5 Data Analysis

#### 2.5.1 Quantitative Analysis

Descriptive statistics (means and standard deviations) were used to summarise participant demographics and baseline characteristics. A repeated measures General Linear Model was used to assess changes in Overall Recovery Capital (Recovery Capital Index) across three time points (baseline, 4 and 12 weeks). For secondary outcomes, a repeated measures multivariate General Linear Model (multivariate analysis of variance) was conducted to examine changes across five variables: Social Recovery Capital, Personal Recovery Capital (two subscales from the ARC), Recovery Group Participation (which is used to measure Community Recovery Capital), Quality of Life and Commitment to Sobriety. Mauchly's test was used to assess sphericity, with Greenhouse–Geisser corrections applied where necessary. All analyses were conducted using SPSS (IBM SPSS Statistics; Version 27).

#### 2.5.2 Qualitative Analysis

Participant interviews were analysed using conventional content analysis [33]. Researchers began with open coding of transcribed data, grouping initial codes into categories based on patterns and relationships between codes. Categories were refined through an iterative review of transcripts to ensure all relevant data were captured. Two authors (A.P. and L.F.) conducted the coding and categorisation, with a third author (A.D.R.) auditing the process to confirm that categories were substantiated from the original dataset. Pseudonyms were used in reporting the qualitative data.

## 3 Results

About 33 people expressed interest in the project, of whom 25 consented to participate. Of these, 21 participants completed the baseline assessment, 15 (71.4%) completed the 4‐week follow‐up and 10 (47.6%) completed the 12‐week follow‐up. Of the 15 participants who did not complete follow‐ups, five did not want to participate further and five were unable to be contacted. For the remaining five participants, no reason for not completing follow‐up was recorded.

The mean age of participants (*N* = 21) was 44 years (SD = 11.1, range 24–66). Eleven participants identified as male and ten as female. The majority reported alcohol as their primary drug of concern (*n* = 15; 71.4%). Demographic characteristics of participants are presented in Table 1.

**TABLE 1:** Participant demographic information.

Variable	Count
*N*	21
Age (mean, SD)	44.0 (11.1%)
Gender	
Man	11 (52.4%)
Woman	10 (47.6%)
Primary drug of concern	
Alcohol	15 (71.4%)
Methamphetamine	4 (19.1%)
Cannabis	2 (9.5%)
Country of birth	
Australia	19 (90.5%)
Other	2 (9.5%)
Education level	
Year 11 or equivalent	3 (14.3%)
Year 12 or equivalent	4 (19.0%)
Vocational training/apprenticeship	8 (38.1%)
Degree	6 (28.6%)

Abbreviation: SD, standard deviation.

### 3.1 Quantitative

#### 3.1.1 Primary Outcome

A within‐subjects General Linear Model was conducted to examine changes in ORCS across three time points (baseline, 4‐ and 12‐week follow‐up) among the 10 participants who completed all assessments. Mauchly's test of sphericity was not significant, indicating that the assumption of sphericity was met. The analysis revealed a significant overall effect of time on recovery capital, *F* (2, 18) = 4.64, *p* = 0.024 where baseline had the lowest recovery capital (*M* = 42, SD = 19), 4 week was higher than baseline (*M* = 57, SD = 18) and the 12‐week follow up was the highest (*M* = 61, SD = 26). The means and standard deviations for recovery capital for participants who completed baseline only, baseline and 4‐week follow‐up and all three time points are shown in Table 2.

**TABLE 2:** Mean Overall Recovery Capital Scores for participants who completed baseline only, two time points and three time points.

	*n*	Baseline, *M* (SD)	4‐week, *M* (SD)	12‐week, *M* (SD)
Baseline only	21	33.9 (19.3)	—	—
Baseline and 4 weeks	15	36.9 (20.8)	51.0 (21.1)	—
All three timepoints	10	42.1 (18.5)	57.2 (18.4)	60.8 (25.9)

This finding was further supported by a paired‐samples *t*‐test comparing baseline and 4‐week recovery capital scores among the 15 participants who completed these two time points, which also showed a significant improvement from baseline (*M* = 37, SD = 21) to 4 weeks (*M* = 51, SD = 21), *t* (14) = −3.36, *p* = 0.005 (see Table 3).

**TABLE 3:** Paired sample *t*‐test for each outcome variable between baseline and 4‐week follow‐up.

Outcome	Time	Mean	SD	*t*	*p*
Overall Recovery Capital Score	Baseline	36.9	20.8	**−3.359**	**0.005**
	4 week	51.0	21.1		
Social Recovery Capital	Baseline	16.3	4.7	**−2.332**	**0.035**
	4 week	18.5	5.2		
Recovery Group Participation	Baseline	0.9	1.6	−1.692	0.113
	4 week	2.4	4.0		
Personal Recovery Capital	Baseline	16.5	5.5	−1.574	0.138
	4 week	18.9	6.8		
Quality of Life	Baseline	12.8	4.0	**−2.409**	**0.030**
	4 week	14.2	3.7		
Commitment to Sobriety	Baseline	23.8	7.2	−0.087	0.932
	4 week	23.9	6.9		

*Note: N* = 15. Bolded lines are significant at 0.05.

Abbreviation: SD, standard deviation.

#### 3.1.2 Secondary Outcomes

Five subscales from the REC‐CAP assessment (Social Recovery Capital, Recovery Group Participation, Personal Recovery Capital, Quality of Life and Commitment to Sobriety) were examined using General Linear Modelling to explore whether any of these subscales contributed to overall changes in the ORCS. However, due to the small number of participants who completed the 12‐week follow‐up (*n* = 10), the overall multivariate test of these five variables was not statistically significant, indicating that a larger sample is needed to identify which sub‐scales most strongly influence changes in ORCS. As a result, follow‐up univariate analyses across the three time points were not conducted. Nevertheless, individual subscales were analysed using *t*‐tests between baseline and the 4‐week follow, which had a larger sample size (*n* = 15). The results indicated significant improvements in Social Recovery Capital and Quality of Life between baseline and the 4‐week follow‐up (Table 3).

Participants set goals that included attending support groups such as SMART Recovery, Alcoholics Anonymous or Narcotics Anonymous; reducing alcohol use by designating alcohol‐free days and avoiding alcohol purchases; improving relationships with partners and children; engaging in physical activity through gym sessions, fitness classes, or walking; and beginning volunteer work.

### 3.2 Qualitative

Interviews were conducted with 6 of the 10 participants who completed the 12‐week data collection and three project staff members (peer workers or intake workers). Conventional content analysis identified two key categories: (i) support for goal setting; and (ii) participant engagement with the peer worker.

#### 3.2.1 Support for Goal Setting

Participant experiences of the intervention were focused on the support provided for setting recovery‐oriented goals while on the waiting list. Michelle (woman, aged 54) shared that the tool ‘helped with my life goals, what I can do today and concentrating on the future’. She added, ‘… between the second and third sessions has been hugely helpful in keeping my mind on not drinking. And we've had a few issues happen this month, so it's been really good keeping focused on staying sober’.

Daniel (man, aged 39) also reflected on his evolving goals throughout the intervention:

… one of the goals [was] to have four NA [Narcotics Anonymous] sessions before [the] next session, and that was four weeks apart … I said, I want to be able to go to the pub, have one beer and stop … I did 20 NA sessions and I didn't have a beer …

Daniel went on to describe the lasting impact of the intervention: ‘the good thing is I didn't relapse or anything like that. I went 101 days without drinking or using coke or anything like that, and literally turned my life around’.

Participants emphasised how support from peer workers complemented the REC‐CAP tool. Nicole (woman, aged 52) initially struggled to articulate her goals, but the peer worker's guidance was invaluable:

I just found it really hard to put into words what it was that I was trying to achieve with my goals, other than, stay sober … but [peer worker] helped me. We talked through some stuff, and then he basically reworded, I guess, what I was trying to say, to put in there.

Setting goals with peer workers also fostered a sense of accountability. Thomas (man, aged 29) reflected:

It was good to have someone to touch base with, to kind of ground me, I guess, and set goals and be held accountable … I had tried in the past for about a year just to do things on my own and wasn't getting anywhere. Just coming to something made a difference.

Daniel echoed this sentiment, linking accountability to the peer worker's ongoing involvement, with ‘check‐ins every couple of weeks and with the sessions I had with him, he helped me be accountable and because he was such a good guy, I didn't want to let him down’. By the end of the intervention, Daniel chose to remove himself from the counselling waiting list, stating: ‘I don't need [treatment] now because I've got what I needed out of it from [peer worker] … I'm on top of everything’.

Peer workers were initially hesitant about the REC‐CAP tool, particularly its fit with their relational model of support. One peer worker noted: ‘sometimes I feel like [clients] look at [REC‐CAP] but they'll just create goals on what they want to create goals on anyway, without really taking the [REC‐CAP] into account’. However, upon further reflection, the peer worker considered a shift in practice: ‘I started thinking about maybe on my part I could do a bit more motivational interviewing and really sort of get to the bottom of what they're wanting and I think that's been beneficial as well’.

Another peer worker appreciated the visual nature of the tool: ‘it's great to be able to see the areas in which they need support … it was really good to have that [visual display] to sort of show them where they're at’. However, despite its benefits, this peer worker also identified challenges, particularly the time required to administer the tool: ‘the length of time that it kind of takes is the biggest barrier because once you've gone through maybe the first half of the questions, their head's kind of beginning to go out the door’. The peer workers identified engagement as another concern, especially for participants who were ambivalent:

For the people who have got one foot in, one foot out, it's not gonna work. Like anything, doesn't matter what you do, it's just not gonna happen … we're making the same SMART goals, because we haven't been able to move in the ones that we've set.

#### 3.2.2 Participant Engagement With the Peer Worker

Participants identified their relationship with the peer worker, beyond the collaborative use of the REC‐CAP tool, as a central benefit during their time on the waiting list. For Nicole, the peer worker's openness and lived experience were inspiring:

Having gone through similar struggles, emotions, challenges, et cetera as me, it was very relatable, and it just really helped reinforce that what I was doing was valid and on track. And having seen his success story … it was just really hopeful and relatable and relevant.

Peer workers also reflected on how the REC‐CAP tool helped facilitate meaningful conversations about recovery. One described how sharing their own lived experience during the meetings section motivated a participant to engage more actively:

I had one guy do that, and he said, like, it's quite funny. He was going through the meetings [section of REC‐CAP]. He's like, you didn't actually do that shit, do you? And I'm like, well, yeah, I do, I've been going to those meetings for four years and then he came back a month later and he'd been going to the meetings every day and he loved it … now he got over 100 days recently and he's doing really well … maybe something he wouldn't have thought about.

A participant, Nicole, described how engaging with the peer worker opened her mind to attending Alcoholics Anonymous:

… I never thought about doing [Alcoholics Anonymous] until I actually saw [peer worker] and he told me how much it will benefit … I was always narrow minded about things like that, but that's the thing that's actually saving me now.

Participants described how engagement with the peer worker transformed their experience of being on the waiting list. Rather than feeling passive or disconnected, they described the intervention as providing structure and purpose during this period. Michelle described it as a ‘focus’ rather than simply ‘waiting for somebody to get in touch with me’. Similarly, Thomas appreciated the immediacy of support:

It was a lot better than just being told, ‘yeah, you'll speak to someone in three months’, and then just being left alone, which, you know, who knows what would happen … to start some program immediately and just, kind of, when I was in that mindset of wanting to make a change that I could then do, as opposed to having to put it on pause for three months.

## 4 Discussion

This study examined the impact of a peer intervention using the REC‐CAP tool to identify recovery strengths and barriers and build recovery capital among individuals awaiting formal addiction treatment. Findings demonstrated a statistically significant improvement in overall recovery capital at both 4‐ and 12‐week post‐baseline, indicating that participants made meaningful gains in the resources that support recovery while still on the waitlist.

These findings align with a growing body of literature demonstrating the positive impact of peer support in enhancing recovery‐related outcomes, particularly during the early stages of engagement. Indeed, one of the participants highlighted the importance of receiving peer support whilst on a wait‐list and in the ‘mindset of wanting to make a change’, underscoring the value of timely, recovery‐oriented contact. Consistent with this, a systematic review of Peer Recovery Support Services by Eddie et al. [34] found that such interventions significantly improve treatment engagement and retention, with additional benefits on personal empowerment and community connection. Peer support has also been found to be effective in increasing abstinence, treatment satisfaction and quality of life in both group and one‐on‐one settings [18, 34, 35, 36], as well as being cost‐effective [37, 38]. Indeed, the presence of other people in recovery in an individual's social network has been shown to be critical during the early stages (initial 12 months) of recovery [39]. The qualitative findings from this study suggest that peer workers contribute to this process in two key ways—as positive social role models and sources of connection, as well as conduits to PBRS groups and networks.

While subscale‐level analyses of recovery capital domains did not show statistically significant change, likely due to the small sample size, the qualitative data provided valuable insight into the mechanisms through which peer support may enhance recovery by building social capital and supporting engagement with community recovery resources. Participants identified the REC‐CAP tool as a helpful tool for goal‐setting and monitoring progress, particularly during the waiting period for formal treatment. Participants described how the tool helped them articulate and pursue meaningful goals. The REC‐CAP tool was not only instrumental in structuring recovery efforts but also fostered a sense of accountability, especially when paired with regular check‐ins from peer workers. By showing growth in strengths and resources, it also offers evidence that the waiting time is not simply a period of stasis but can help build recovery engagement and motivation.

One of the six participants interviewed reported that they no longer required formal counselling following the intervention. This outcome, consistent with White's [40] assertion that recovery should precede treatment, highlights the potential of such interventions to reduce overall wait times by equipping individuals with early access to support, strengthening their coping mechanisms and addressing concerns before escalation to clinical services is necessary. In doing so, the intervention may not only ease demand on specialist care but also underscore the therapeutic value of peer relational approaches in promoting recovery and self‐efficacy [35]. This aligns closely with the principles of the stepped care model, wherein individuals progress through increasingly intensive interventions based on need, beginning with the least resource‐intensive options [41], and where peer recovery support can constitute a form of prevention or early intervention.

Beyond the REC‐CAP tool, participants identified the relationship with the peer worker as important to their intervention experience. The peer workers' lived experience, openness and encouragement were described as deeply validating and motivating. The peer worker's influence also extended to tangible behavioural changes, such as initiating attendance at peer support meetings, highlighting the power of peer modelling and relational trust in activating recovery capital. These findings reinforce the importance of peer support in bridging gaps in service access and sustaining motivation during critical periods of recovery. The findings also highlighted the credibility of peer workers who have ‘real world’ experience and are consistent with previous findings that peer referrals to support groups are more effective than referrals from clinicians [42].

These findings should be interpreted with caution given the small sample size, participant attrition and absence of a control group such that the observed improvements in recovery capital may, in part, reflect the effects of the treatment received and cannot be attributed solely to the intervention alone. In addition, it was not possible to separate intervention effects from those of standard treatment for people who had shorter durations on the waiting list. The inclusion of additional feasibility measures, such as assessments of intervention fidelity to determine the extent to which peer workers delivered the intervention as intended, would have further strengthened the evaluation. Nonetheless, the significant improvement in overall recovery capital, reinforced through the qualitative interviews, provides preliminary support for the intervention's potential to strengthen recovery resources. Larger controlled trials are needed to more rigorously examine these effects over time and across specific recovery domains.

## 5 Conclusion

Peer support and care planning show promise in increasing recovery capital and maintaining motivation during treatment delays for people waiting for alcohol and other drug treatment. Participants valued the peer relationship and the opportunity to reflect on recovery goals. These findings support further investigation in a fully powered trial to assess impact, effectiveness and scalability.

## Author Contributions

Each author certifies that their contribution to this work meets the standards of the International Committee of Medical Journal Editors.

## Funding

This work was supported by the Helen and David Hains Foundation. D.I.L. is supported by a National Health and Medical Research Council Investigator Grant (#1196892).

## Conflicts of Interest

The authors declare the following financial interests/personal relationships which may be considered as potential competing interests: Victoria Manning reports a relationship with the National Health and Medical Research Council, VicHealth, Victorian Government Department of Health, Victorian Responsible Gambling Foundation, National Centre for Clinical Research on Emerging Drugs and HCF Research Foundation that includes funding grants. Victoria Manning is an academic editor of Drug and Alcohol Review. David Best reports a relationship with Scottish Recovery Consortium, Newcastle City Council, Birmingham City Council, Recovery Connections, Indivior, All Rise, ARCHway Institute, Clean Cause Foundation and R1 Learning that includes: consulting or advisory. Dan I. Lubman reports a relationship with the National Health and Medical Research Council and Camurus that includes: funding grants. The other authors declare no conflicts of interest.

## Supporting information


**Appendix A.** The Template for Intervention Description and Replication (TIDieR) Checklist.

## Data Availability

The data that support the findings of this study are available from the corresponding author upon reasonable request.
